# Predictive Analytics of Cattle Host and Environmental and Micro-Climate Factors for Tick Distribution and Abundance at the Livestock–Wildlife Interface in the Lower Okavango Delta of Botswana

**DOI:** 10.3389/fvets.2021.698395

**Published:** 2021-10-28

**Authors:** Nlingisisi D. Babayani, Anastacia Makati

**Affiliations:** Okavango Research Institute, University of Botswana, Maun, Botswana

**Keywords:** micro-climate, environment, host, ticks, wildlife-livestock, model, abundance, distribution

## Abstract

Tick distribution and abundance is influenced by several factors that include micro-climate and environmental and host factors. Contextual understanding of the role played by these factors is critical to guide control measures. The aim of this study was to determine the predictive value of these factors for changes in tick distribution and abundance at the livestock–wildlife interface of the lower Okavango Delta. A two-stage quota sampling design was adopted to select 30 clusters of seven cattle each for tick investigation. Tick investigation was done by lifting the tail to count the total number of ticks at the anno-vulva region during the four meteorological seasons of the year. Additional data were collected on ear tag number, location of origin, sex, age, body condition score (BCS), season of the year, stocking density, and Normalized Difference Vegetation Index values of source terrain. A random effects model was used to evaluate predictive value of the above for tick abundance. Additional mapping of tick distribution pattern in the abattoir catchment area was conducted using spatial autocorrelation and hot-spot analysis. Tick intensity of infection increased linearly from males to females (*Z* = 3.84, *p* < 0.001), decreased linearly from lower to higher BCS (*Z* = −4.11, *p* < 0.001), and increased linearly from cold-dry through dry to wet seasons (*Z* = 10.19, *p* < 0.001). Significant clustering of neighboring crushes on account of tick count was noted in the late-hot-dry season, with high tick count in crushes located along the seasonal flood plains and low tick counts in those located in the dry grasslands. It was concluded from this study that cattle tick abundance is influenced largely by season of the year and that the micro-climatic conditions brought about by the seasonal flooding of the delta have a decided effect on tick distribution during the driest of the seasons. Our study has, for the first time, profiled drivers of tick distribution and population growth in this unique ecosystem. This has the potential to benefit human and veterinary public health in the area through implementation of sustainable tick control strategies that are not heavily reliant on acaricides.

## Introduction

Ticks are among the most important global vectors of infectious pathogens of humans and animals (1). The rising global incidence of tick-borne diseases (TBDs) is indicative enough of the significant threats to human and veterinary public health posed by ticks and the diverse infectious pathogens they transmit (2). The direct and indirect negative impact of tick infestation to domestic animal hosts is well-documented and ranges from significant production losses that includes meat, milk, eggs and hides, to deaths of the affected animals through blood loss and tissue invasion by secondary microbes as well as pathogens they themselves transmit during feeding. In addition, there are economic losses associated with the control of ticks, treatment of clinical cases, and unearned income owing to production inefficiencies, with a global annual estimated costs of between US$ 13.9 and US$ 18.7 billion in cattle alone (3).

Changes in distribution and abundance of ticks, as well as pathogen transmission dynamics are influenced by several factors such as micro-climate and climate conditions (4); host factors like availability, suitability, and density (5); and direct and indirect anthropogenic factors (6). An understanding of the role played by these factors not only helps guide adoption of evidence-based husbandry management practices to sustainably control against ticks in domestic animals but also is critical in the implementation of sound public safety guidelines aimed at guarding against human infestation and potential debilitating tick-borne pathogens (TBPs) they transmit.

Information on available ticks, their temporal and spatial distribution, and determinants thereof, as well as disease risk they present to humans and animals, is scant and grossly limited in Botswana. For instance, mortality of more than 16,690 cattle, 18,160 goats, 1,900 horses, 1,385 donkeys, and 559 sheep was reported by 504 farmers from 2015 to early 2019 in the lower Okavango Delta areas (7). Although these figures were near impossible to ascertain owing to the extensive nature of cattle farming and the suspicion that farmers may have exaggerated the figures to motivate for some form of relief from the government, anecdotal evidence from clinical case response to treatments indicated widespread infections with TBDs, mainly tick-associated Heartwater disease (caused by *Ehrlichia ruminantium* infection, transmitted by *Amblyomma* spp. ticks) and secondary infection with Dermatophilosis (caused by *Dermatophilus* spp. infecting *Amblyomma* spp. tick bites). Such evidence is not only limited to livestock infections but humans too. More than 30% of the 169 US Army soldiers deployed in Botswana for 2 weeks in January 1992 for a field training exercise developed a febrile illness within 5 days of their return, suggestive of infection with TBDs (8).

The predictive value of host, environmental, and micro-climatic factors for tick distribution, abundance, and infestation levels of cattle in the lower Okavango Delta is unknown. This is so despite the uniqueness of the ecosystem in that it is an intense interaction interface between wildlife and domestic animals (9) and, hence, potential for ticks and tick-borne diseases (TTBDs) transmission between livestock and sympatric wildlife. Also, the area receives seasonal floods from the delta that arrives during the dry season, hence likely to influence tick life cycle along the flood plains. Paucity of information on TTBDs in the face of heightened threats to human and veterinary public health from such can only lead to deleterious effects on animal welfare, human well-being, and rural livelihoods. In this study, we attempted to determine the predictive value of environmental and micro-climatic factors on changes in the distribution and abundance of ticks in general at the livestock–wildlife interface of the lower Okavango Delta areas by investigating cattle reared in the study area and destined for slaughter for ticks at the local abattoir, as an indicator species for domestic stock in the area. We predict that tick abundance (measured as intensity of infestation on cattle) is associated with vegetation indices and season. It is hoped that such a model will be of value in forecasting risk of TTBDs by highlighting critical indicators. This will be of particular importance to occupational groups like farmers, agricultural workers, slaughterhouse workers, and veterinarians who are known to experience a riskier potential for TBP transmission (10).

## Materials and Methods

### Study Area

**Figure 1:**
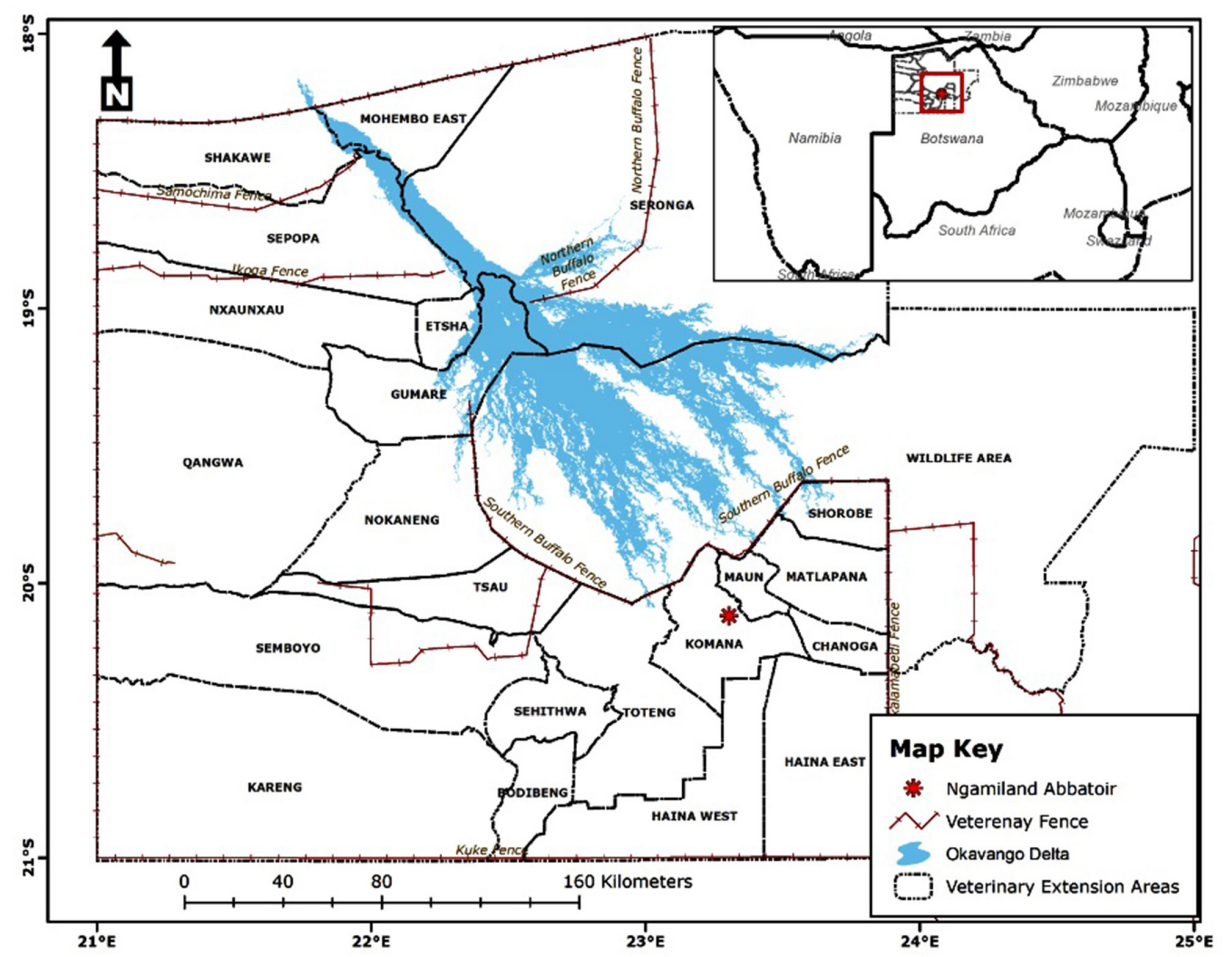
Location of Ngamiland abattoir and catchment areas for supply of cattle for slaughter [Source: Adapted from the Department of Veterinary Services' Veterinary Epidemiology and Economic Section (VEES), Botswana, 2020].

**Figure 2:**
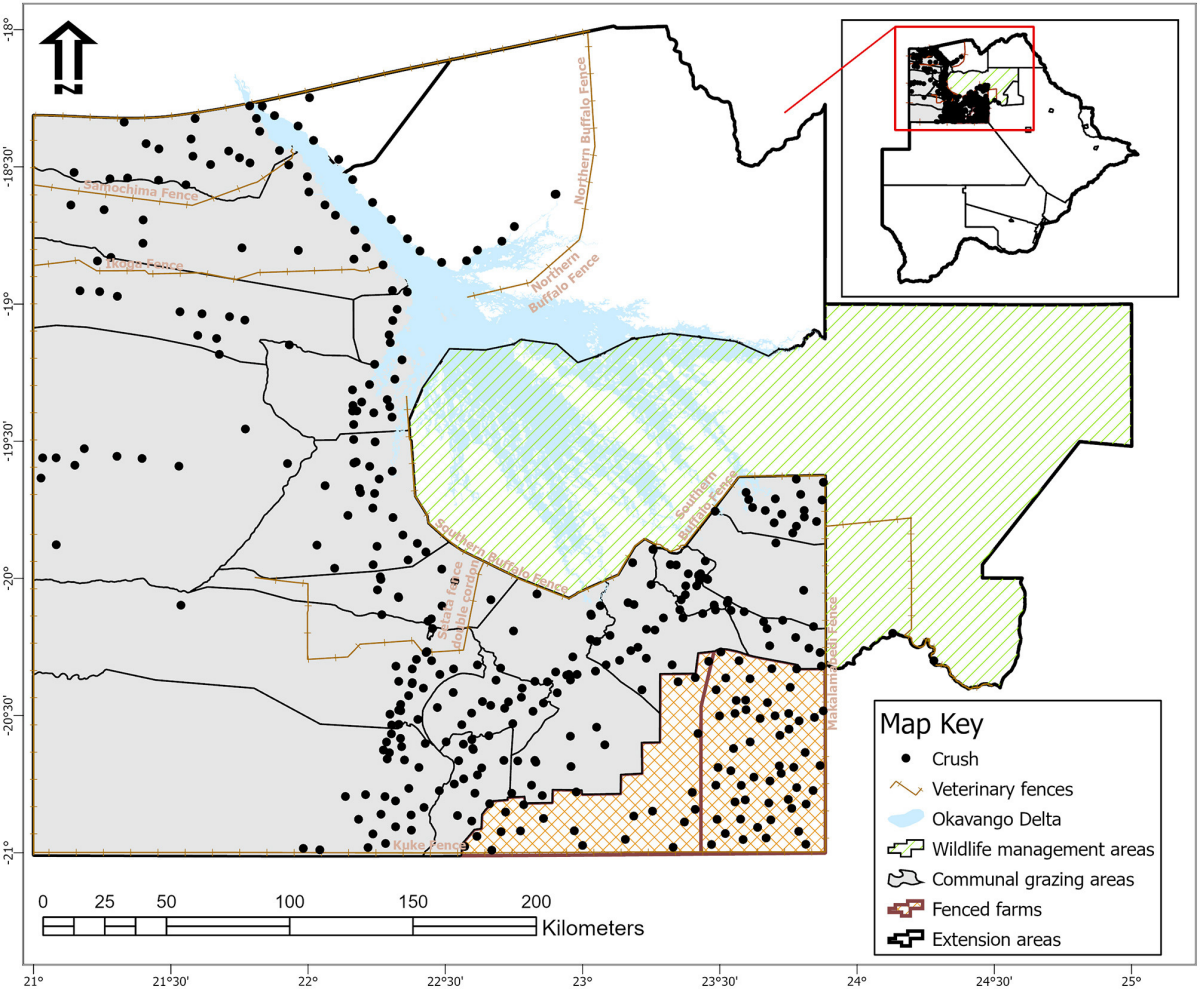
Map of Ngamiland abattoir catchment areas showing location of veterinary fences, crushes, and their spatial relation to wildlife management areas [Source: Adapted from the Department of Veterinary Services' Veterinary Epidemiology and Economic Section (VEES), Botswana, 2020].

The study was conducted at Ngamiland abattoir (20° 7′ 11.09″ S, 23° 18′ 18.61″ E), Northwest District of Botswana, on cattle supplied from its traditional catchment areas and the large extent of that area interface with the lower Okavango Delta for approximately 240 km along the southern buffalo fence (Figure 1). Catchment area for the abattoir is subdivided into six subzones through manmade veterinary cordon fences (11, 12) aimed at restricting livestock movement to limit spread of transboundary animal diseases. Collectively between the sub-zones, there are about 192,000 cattle [Department of Veterinary Services (DVS) foot-and-mouth disease vaccination campaign data, 2020, unpublished] and 80,000 small ruminants (sheep and goats) (13), with density highs of 100 livestock unit per square kilometer along the lower Okavango Delta (14). High concentration of livestock along the lower Okavango Delta is attributed to better grazing and water availability from seasonal flooding of the delta. Cattle in the catchment areas of the abattoir, except for those in the southern parts, which consist of fenced farms, are reared under an extensive husbandry management system that is centered around communal grazing rights with minimal herding. Small ruminants in these areas are largely concentrated around homesteads for close monitoring against predators. On the other side of the southern buffalo fence is the Okavango Delta, a wildlife management area with abundant and concentrated variety of wildlife species that includes large herbivores like elephants and buffalo (15). Large herbivores, particularly elephants, do trample down and create breakages on veterinary fences that cross their seasonal migratory routes in this area (16). Breakages on fences that are created by elephants or wear and tear because of maintenance shortfalls allow for livestock to ingress into wildlife management areas and for wildlife to egress to livestock rearing areas. Such incursions allow for contact between wildlife and domestic stock, initially across the southern buffalo fence and continue across subzone barrier fences. Departure point of cattle for supply to the abattoir is the crush, and movement of cattle destined for slaughter at the abattoir is through a permit. A designated crush is formed out of a group of herds that closely mix at pasture and is signified by a central point location where communal cattle handling facilities are located to facilitate routine husbandry management practices. A crush is deemed the smallest epidemiological unit for disease control purposes in extensively managed herds in Botswana and strategic grouping of crushes forms an extension area under an extension official whose mandate is to provide service to farmers in all the aligned crushes (Figure 2).

### Data Collection

A two-stage quota sampling design (30 × 7) (17, 18) was adopted in this study to estimate tick count to within ±10 percentage points of the true proportion, with 95% confidence. Thirty crushes were selected on a first come, first served basis as trucks deliver cattle at the abattoir lairage. All cattle that were delivered at the abattoir were selected for sampling from a consignment with seven or less cattle and originating from a crush not encountered before. If more than seven cattle or the number required to add up to seven from a previously encountered crush are presented, random selection based on ear tag number as a unique identifier was used to pick the number needed from that consignment until seven cattle have been reached. This process was repeated daily until the first 30 different crushes with seven cattle from each is achieved for that season of the year. As per the survey design, seven cattle from 30 crushes makes a sample size of 210 cattle to be investigated for ticks during each of the cold-dry (June/July/August 2019), late-hot-dry (Sept/Oct/November 2019), wet (December 2019/January and February 2020), and early-dry (March/April/May 2020) seasons. The assumption with this methodology was that supply of cattle to the abattoir for slaughter was done with probability proportionate to the size (PPS) of population at the source crush (cluster) such that larger crushes would have most likely supplied cattle and hence a greater chance of being selected among the first 30 crushes in each of the seasons. Repeating the survey every season allowed for replacement so that each crush could be included in the sample more than once in a year (18).

**Figure 3:**
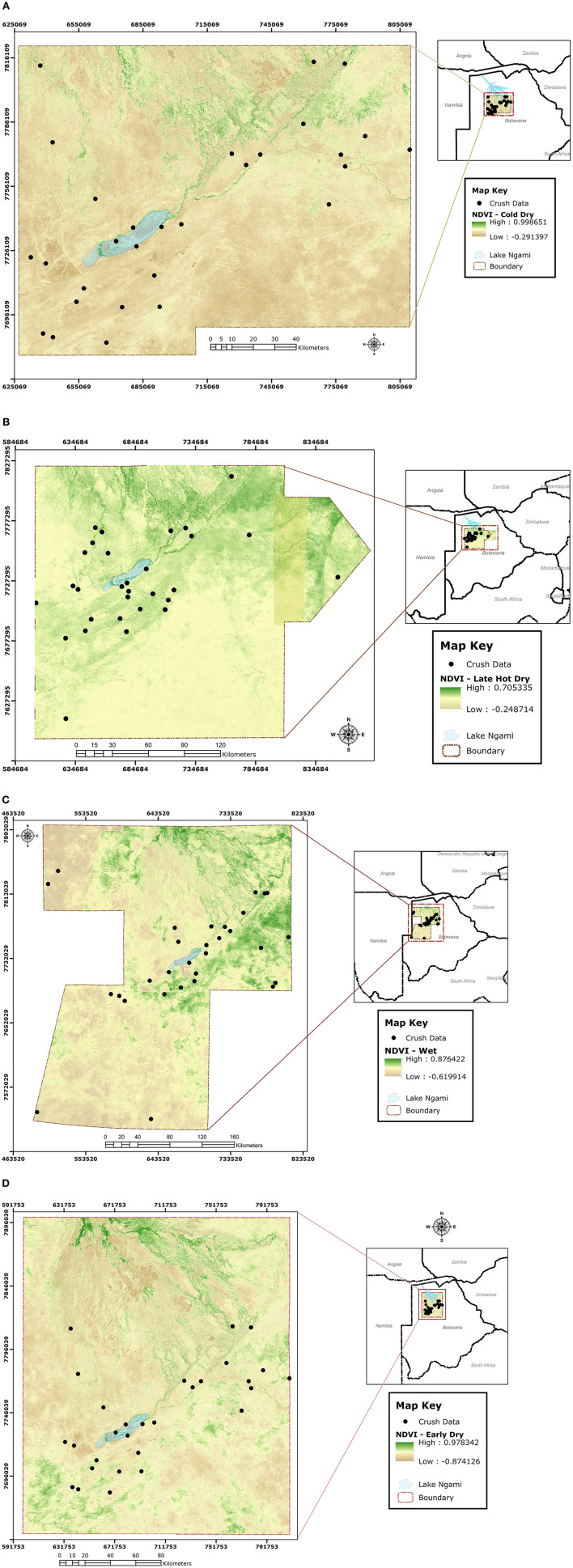
Sentinel 2 satellite images obtained over the study area with superimposed point locations of crushes from which NDVI values were extracted during each of the four seasons, being cold-dry **(A)**, late-hot-dry **(B)**, wet **(C)**, and early-dry **(D)**.

During slaughter, the selected cattle from each crush were investigated for ticks along the lairage corridors leading to the stunning box. This was done by lifting the tail to count the total number of ticks at the anno-vulva region. Although ticks were also collected from the anno-vulva region of selected cattle to point of saturation (19) for each selected crush, only tick counts were considered for this analysis and identification of ticks to species/genus level in cattle reared in the study area was recently done by Raboloko et al. (20). Since our objective was to approximate determinants of spatial distribution and abundance of ticks in general, and not specific tick species, as indicators for infestation risk to cattle reared in the study area, we conducted non-specific counts of all ticks at the anno-vulva region to address it. The anno-vulva region was selected for tick investigation in this study because it is a known preferred site for most ticks in cattle (21). Data on crush and extension area of origin for the selected cattle, ear tag number, sex, body condition score (BCS) on a scale of 1 to 9 adapted from the 1 to 5 scale by (22), and season of the year were collected at the time of tick investigation. Using the ear tag number, additional data on age of cattle, stocking density at extension area of origin, and geo-coordinates of the crush of origin were extracted from the Botswana Animal Identification and Traceability System (BAITS) database (https://www.baits2.gov.bw). The crush geo-coordinates were subsequently used to locate terrain at each source crush and enable calculation of Normalized Difference Vegetation Index (NDVI) values corresponding to the point in time when the cattle were investigated for ticks in each of the four seasons. Briefly, NDVI values were calculated from the 10-m-resolution product (with four bands) of Sentinel 2 satellite (23) image obtained over the study area during each of the four seasons. Using the “extract values to points” tool in ArcGIS 10.8, NDVI values at the exact crush location were extracted from the image obtained for each of the four seasons (Figure 3) and this approach has been used successfully by others before (24, 25).

### Data Analysis

A random effects linear regression model was used to assess the dependency of tick abundance on NDVI, age of cattle in months, stocking density at source extension area, sex of the animal, its BCS, and season of the year it was investigated for ticks. The random effects model was adopted for this analysis to account for the correlated nature of data at crush and extension area levels and the large number of clusters, 17 extension areas, and 96 crushers. This approach ensures that the above cluster variables are included as a random effect to explicitly model the between-cluster variance (26). Sex, BCS, and season of the year were fitted as categorical variables, while extension area and crush were treated as two levels of clustering with crush nested within extension area and the order of nesting proceeded from left to right in the model. An a priori interaction term between season and NDVI was further fitted on the model to investigate the dependency of tick count on NDVI conditioned on season of the year. A test for trend was also conducted for significant categorical predictors in the model that have more than one category to investigate for a dose–response effect. The analysis was done using STATA^®^/IC 10 statistical software. To assess the distribution pattern of ticks, a spatial autocorrelation global statistical test, Moran's *I* index, was performed for each season of the year to determine whether neighboring crushes are similar or dissimilar with respect to tick counts. This was done using tick counts as input field at each crush geo-location and the index's *p*-value was calculated to assess whether any observed clustering of similar tick counts is statistically significant or whether it occurred by chance (27). Mapping of crushes for the season(s) with significant clustering of crushes on the basis of tick counts was done in ArcGIS 10.8.1 spatial analysis software using the hotspot analysis tool in order to identify characteristics of clustering patterns not revealed by the Moran's *I* statistic alone, that is, the degree of clustering for either high or low tick counts values (28–30) across the study area.

## Results

**Figure 4:**
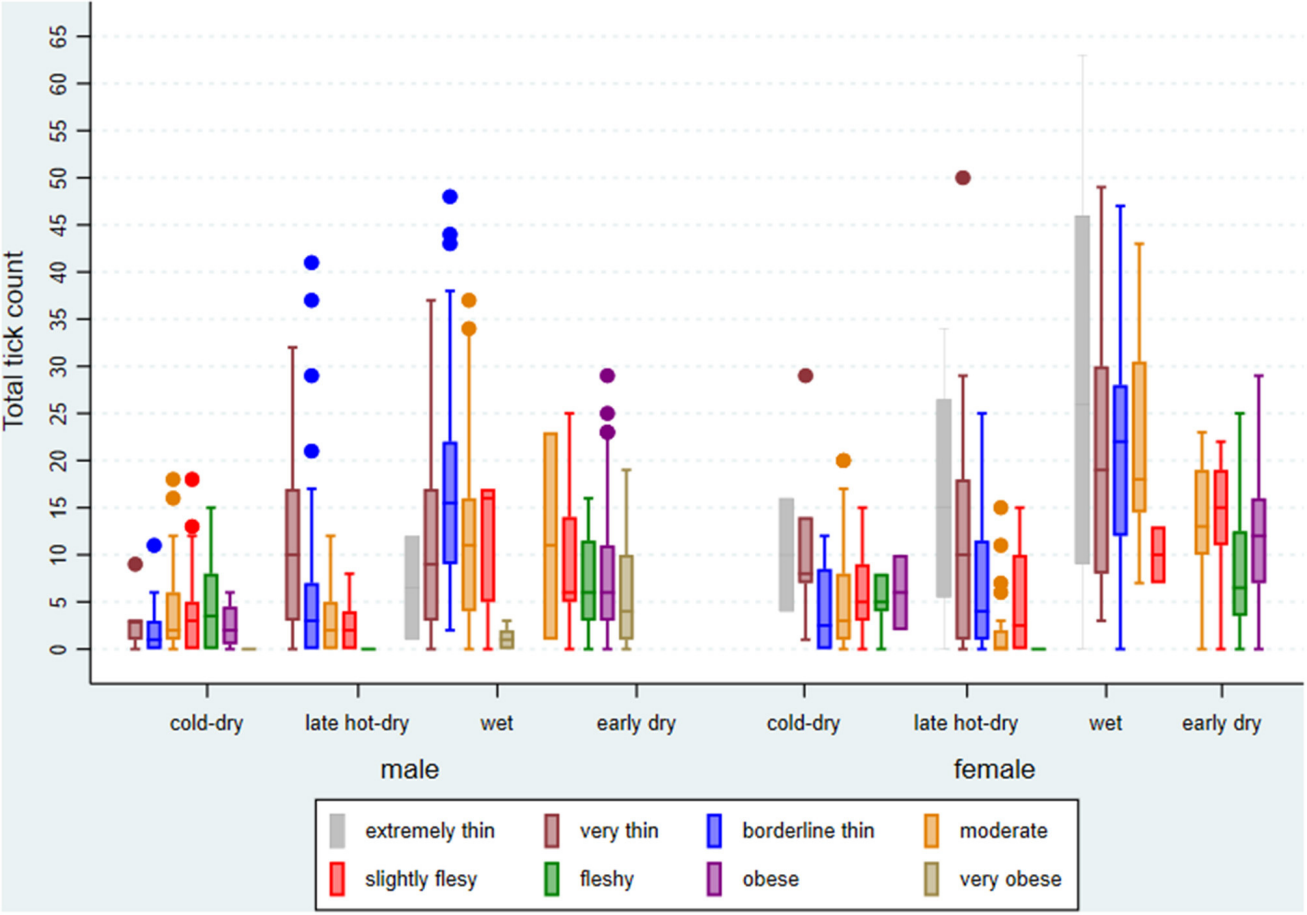
Box plot of summary statistics for tick counts by season of the year, sex, and body condition score (BCS), excluding missing BCS, of cattle investigated at Ngamiland abattoir during the study.

From the sampling plan, about 840 cattle were expected to be investigated for ticks and 832 were done over the four seasons. BCS for the cattle presented for slaughter and investigated during the study ranged from 2 to 9 (median score = 5). The average age was 47 (range 10–181) months. There were 63 (7.57%) cattle with missing records on age, implying that they were tagged and issued with manual permits for slaughter at the abattoir before being entered into the database. Cattle with any missing record were omitted from the analysis. Cattle density at extension area level ranged from 1 to 22 (mean = 7) per square kilometer. The number of ticks counted per animal and per crush ranged from 0 to 63 (mean and standard deviation = 9.04 ± 9.46). However, the counted number of ticks per animal differed by season of the year, BCS, and sex (Figure 4).

The two models, one containing only the full set of main effects predictors and the other containing an additional interaction term were highly significant (Wald χ^2^ = 234.37; df = 14; *p* < 0.001 and Wald χ^2^ = 246.63; df = 17; *p* < 0.001, respectively), indicating a good fit of both models to the data when compared to the null model consisting only of a randomly varying intercept. A test of the null hypothesis of no within-extension area and within-crush correlation vs. the alternative (presence of within-extension area and within-crush correlation) was highly significant for both the main effects and interaction models (χ^2^ = 76.68; df = 2; *p* < 0.001 and χ^2^ = 83.12; df = 2; *p* < 0.001, respectively), indicating data correlation and hence validation for the choice of a random effects model over a simple linear regression model. Coefficient estimates for explanatory variables, their standard errors, and significance levels for both the main effects and interaction models are shown in Table 1. Tick intensity of infection increased linearly from males to females (*Z* = 3.84, *p* < 0.001), decreased linearly from lower to higher BCS (*Z* = −4.11, *p* < 0.001), and increased linearly from cold-dry to late-hot-dry through wet seasons (*Z* = 10.19, *p* < 0.001).

**Table 1:** Random effects results for the dependency of tick count at the anno-vulva region of cattle brought for slaughter on select predictor variables, with age in months, stocking density at source extension area, sex with male as the baseline, body condition score (BCS) with BCS = 2 as baseline, Normalized Difference Vegetation Index (NDVI), and seasons of the year with cold-dry season as the baseline.

	**(1)**	**(2)**
**Variables**	**Mean tick count for the main effects model**	**Mean tick count for the interaction model**
Age in months	0.00191 (0.0116)	0.000353 (0.0115)
Stocking density	0.243 (0.138)	0.253 (0.150)
Female	2.561*** (0.596)	2.472*** (0.595)
BCS = 3	−4.426** (2.143)	−4.603** (2.133)
BCS = 4	−4.994**(2.098)	−5.071**(2.090)
BCS = 5	−7.202*** (2.158)	−7.265*** (2.149)
BCS = 6	−6.739*** (2.267)	−7.050*** (2.263)
BCS = 7	−9.993*** (2.404)	−10.16*** (2.398)
BCS = 8	−10.38*** (2.492)	−10.72*** (2.488)
BCS = 9	−16.22*** (2.969)	−16.47*** (2.958)
Late-Hot-dry season	−0.0833 (1.116)	11.13*** (3.851)
Wet season	9.463*** (1.215)	11.45*** (3.478)
Early-Dry season	6.261*** (1.197)	12.99*** (3.627)
NDVI	1.033 (6.233)	18.60 (12.18)
NDVI conditioned on late-hot-dry-season		−60.09*** (19.56)
NDVI conditioned on wet season		−11.81 (13.52)
NDVI conditioned on early-dry season		−29.50** (14.69)
Constant	9.115*** (3.010)	5.469 (3.659)
Observations^a^	769	769
Number of groups	17	17

*Results marked (1) are from the main effects model and those marked (2) are from the interaction model*.

*Standard errors in parentheses*

*** *p < 0.01*,

** *p < 0.05*.

a *Not including the 63 cattle with missing age records*.

**Figure 5:**
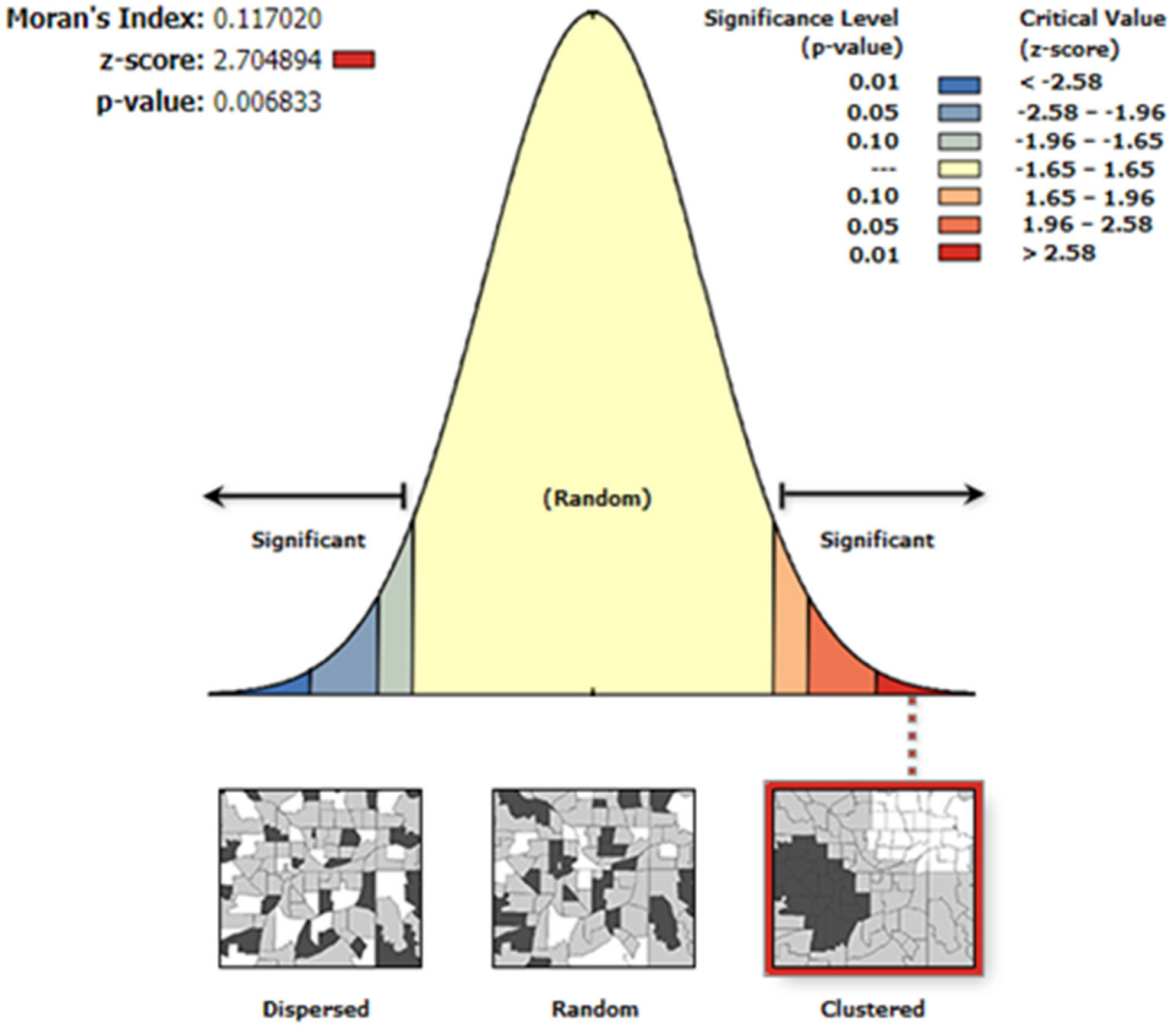
Autocorrelation analysis results for the late-hot-dry season showing a <1% likelihood that the similarity in tick counts between neighboring crushes could be a result of random chance and, hence, significant.

**Figure 6:**
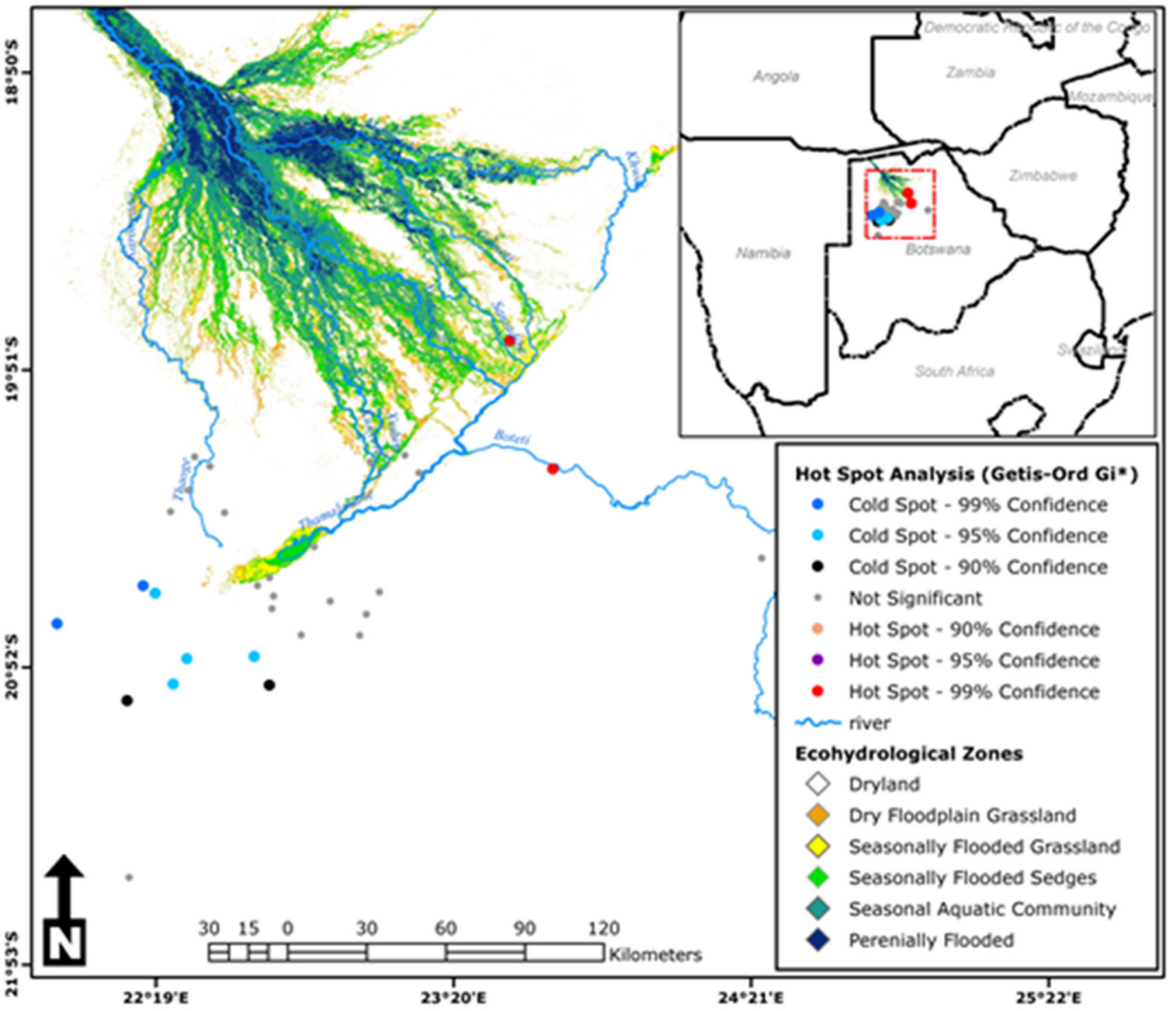
Hot-spot analysis results for the late-hot-dry season showing cluster patterns of tick counts between neighboring crushes across the study area [Source: Adapted from Murray-Hudson et al. (52), Wolski et al. (53), and Inman and Lyons (54) and Landsat 5, 7, and 8: 1990–2019; ODIS].

Moran's *I* index was not significant for cold-dry (*Z* = −0.047, *p* = 0.929), wet (*Z* = 0.002, *p* = 0.618), and early-dry (*Z* = −0.082, *p* = 0.707) seasons, hence dissimilarity between neighboring crushes with respect to tick counts during this seasons. For the late-hot-dry season, there was a significant clustering of crushes (Figure 5), indicating similarity between neighboring crushes on account of tick counts. Further mapping of the late-hot-dry season clustering pattern across the study area revealed hot spots (high tick counts) along the seasonally flooded areas of the lower Okavango Delta and cold spots (low tick count) in the dry grassland far removed from seasonally flooded areas (Figure 6).

## Discussion

Insufficient information on TTBDs, particularly on determinants of changes in tick distribution and abundance heightens threats to human and veterinary public health (2). Identification of host, environmental, and micro-climate factors that drives such changes facilitates formulation of evidence-based integrated approaches to tick control and promotes sustainability, particularly when done with a deliberate aim to limit overreliance on acaricides, which resource-poor farmers such as in the current study area can hardly afford. Such an achievement is likely to improve local food security through limited livestock deaths and mitigate against the risk of potential for TBP transmission to occupational groups. In this study, we conducted an abattoir-based serial cross-sectional investigation of ticks at the anno-vulva region of cattle presented for slaughter and used a random effects model to correlate tick counts with a priori host, environmental and micro-climate factors as a means of evaluating their predictive value for measured tick abundance, with additional spatial analysis to determine the distribution of such an abundance.

### Effect of Vegetation Cover on Tick Abundance

NDVI, which is a measure of photosynthetic activity on the ground, was not a significant predictor of tick abundance when modeled as a main effect. However, it was shown to be a significant predictor when conditioned on early-dry and late-hot-dry seasons. Although this positive correlation with TTBDs abundance has been reported previously in literature by others (31, 32), here in the lower Okavango Delta, it appears to fashion a unique situational influence on tick abundance that varies with season of the year. Early-dry and late-hot-dry seasons are seasons of no rain, with the latter coming after almost 6 months of no rain in the country. Available surface water, particularly during the late-hot-dry season, is found only along the flood plain tributaries of the delta resulting in a discernible finger-like green vegetation cover interspaced with dry grasslands. The model analysis shows that this is the time when NDVI has a significant effect on tick abundance. During the wet season, the green vegetation cover and surface water availability is extensive because of the seasonal rains that extend into the cold-dry season leading to dispersal of cattle, ticks passively along, across the entire lower Okavango Delta area. These are the seasons when NDVI has been shown to have no significant effect on tick abundance, and it may highlight the fact that other factors may also drive tick abundance beside cattle, for intance, cattle management practices, density of alternative hosts such as goats, scrub hares, and other wildlife across the study area.

### Effect of Seasonal Patterns on Tick Abundance and Distribution

Significantly more ticks were sampled in the late-hot-dry, wet, and early-dry seasons when compared with the cold-dry season and a dose–response effect was depicted between tick count and season of the year. Significant clustering of ticks was noted during the late-hot-dry season with hot spots (high tick counts) at crushes located in the seasonal flood plains of the Okavango Delta and cold spots (low tick counts) concentration at crushes far removed from the flood plains and located in the dry grasslands. This finding is in agreement with the noted significant predictive value of NDVI during the dry seasons and is consistent with previous findings elsewhere in which variations in micro-scale climatic conditions, including temperature, wind speed, degree of exposure, relative humidity (RH) deficit, and soil moisture near the ground surface, varied the survival and developmental rate of ticks to influence population growth and behavior as well as seasonality of activity (4). The flood plains of the Okavango Delta consist of a deciduous forest that loses its leaves during dry seasons and becomes the only source of permanent water pools from the seasonal floods during the late-hot-dry season. This presents a more shielded habitat with permanent leaf litter layer, providing a uniform microclimate that benefits the development and hence abundance of ticks when compared with dry grasslands (5, 33). Both hard and soft ticks have narrowly defined temperature and humidity deficit ranges for development, activity, and survival (34, 35). Temperature extremes and desiccation has a differential impact depending on species, developmental stage, sex, age, and physiological condition (36). Temperature and humidity vary with meteorological seasons; hence, ticks also show seasonal variation in activity, as seen in our study, and such variation differs between species and developmental stages (37). Congregation of cattle and other alternative hosts that includes wildlife along flood plains to access better grazing and water during the driest of the seasons, coupled with existing suitable micro-climatic conditions to promote tick growth, is judged to precipitate clustering of ticks through passive dispersal toward the plains and host availability along the plains.

### Effect of Host Factors on Tick Abundance and Distribution

A dose–response effect was depicted between tick count and sex as a linear increase from males to females, and BCS as a linear decrease from lower to higher scores. A statistical significant difference in tick count between cattle with poor and good body condition, as found in this study, has also been reported by Tadesse et al. (21) and Eyo et al. (38). Although a cross-sectional study cannot prove causality because of the simultaneous measure of exposure and outcome at a point in time (39), the reduced tick counts in individuals with higher BCS may indicate the negative impact of tick infestation on weight gain (40) or an increase in susceptibility of individuals with low BCS to infestation due to factors such as reduced immune responses and reduced histamine-stimulated grooming (41, 42) owing to negative energy balance (43).

The finding of high tick counts on female cattle when compared to males may indicate the influence of anthropogenic factors that includes trade and husbandry practices (6, 44, 45), as well as probable stimulation of species-specific molecular mechanisms that are sex dependent (46). Besides trade, female cattle are kept for milking by pastoralist throughout the study area (47); hence, they have greater demands (lactation) on their limited resources and are kept closer to human dwellings than male cattle that are rounded off once in a while from extensive grazing pastures for selection to trade. Farmers in the study area rear other animals besides cattle, which include pets and small ruminants, which may act as reservoirs for cattle infection with ticks and impacting female cattle more when compared to male cattle that rarely come close to dwellings. The low cattle density recorded across the expansive extension areas of the study area and the probable low susceptibility of indigenous cattle to tick infestation, in the face of other alternative reservoir species that includes wildlife for multi-host tick species, may have been the host factors that resulted in the dissociation between tick count and cattle stocking density (48). Age was not a suitable predictor of ticks in our study even though it has previously been found to be elsewhere by others (49). This may be attributed to the non-representativeness of age in our study in that cattle brought for slaughter are mainly adults of a certain age category as the price at the abattoir is based solely on weight and hence not representative of the source population age ranges.

### Study Limitations and Way Forward

Although cattle within crushes were randomly selected for sampling at the abattoir, selecting cattle to send for slaughter from the source population is hardly a random process. Employed selection criteria are biased in that it is mainly aimed at maximizing returns from sales at the abattoir and such an undertaking has the potential to select for or against the very attributes we set out to measure in our study and hence likely to unduly influence the study outcomes.

For convenience reasons, only the anno-vulva region was investigated for ticks in this study, which may bias results toward *Rhipicephalus* spp. and *Hyalomma* spp. (50) and may underrepresent the true levels of tick infestation as some tick species have other preferred sites on cattle (21). However, this is not expected to have had a significant impact on the results of this study as *Rhipicephalus* spp. and *Hyalomma* spp. are highly abundant in this region (20, 51), and the focus of this study was the influence of relative differences in tick counts between the crush epidemiological units.

Follow-up studies from this work are needed to identify the tick species found in cattle by spatial locations and probable pathogens they harbor. This will be of crucial importance to evidently estimate the occupational risk of abattoir works to TBDs as it has been shown in this study that most cattle presented for slaughter at the local abattoir in any season of the year carried ticks at the anno-vulva region, with an infestation prevalence in cattle of 73.4% (20) having been established before in the same study area.

## Conclusions

We have shown in our study that cattle tick abundance is influenced largely by season of the year and that the micro-climatic conditions brought about by the seasonal flooding of the delta have a decisive effect on tick distribution during the dry seasons. This was evidenced by the increase in tick counts across the study area during the wet season of the year when compared to the cold-dry season and an overall decrease in tick counts from the early-dry season to lowest counts during the cold-dry season with a steady rise from the late-hot-dry season. Additionally, the rise in tick counts during the late-hot-dry season was characterized by a significant clustering of low tick counts in the dry grassland areas and of high tick counts along the seasonally flooded areas of the Okavango Delta. The clustering of high tick counts in the flood plains is most likely influenced by the suitable micro-climate environment created by seasonal flooding of the delta and hence increased survival and developmental rate of ticks leading to population growth. Furthermore, the significant predictive value of NDVI noted during the early-dry and late-hot-dry seasons supports the narrative of suitable micro-climate for growth and survival of ticks along the flood plain environments during the driest of the seasons. Also, passive dispersal (5) of majority of ticks toward the flood plain areas and away from the dry grasslands during the late-hot-dry season may be a possibility as concentration of tick hosts, both wildlife and livestock, increases along flood plains during the driest of the seasons to access better grazing and drinking water. Our study has, for the first time, attempted to characterize likely host, environmental, and micro-climatic drivers of tick distribution and population growth in the lower Okavango Delta areas of the country. Such knowledge will be of value in forecasting risk of TTBDs through measurement and monitoring of critical indicators of value as identified during this study to warn and protect at-risk groups. Additionally, our results are expected to improve extension service to farmers by guiding implementation of sustainable tick control strategies that are not heavily reliant on acaricides use as well as benefit further research on ticks in the area, particularly on when and where to target environmental sampling of ticks for better dividends.

## Data Availability Statement

The raw data supporting the conclusions of this article will be made available by the authors, without undue reservation.

## Ethics Statement

The animal study was reviewed and approved by University of Botswana Institutional Review Board. Written informed consent was obtained from the owners for the participation of their animals in this study.

## Author Contributions

NB conceived and designed the study, collected field data, conducted its analysis, and drafted the initial manuscript. AM collected desktop spatial data and conducted the spatial analysis. NB and AM contributed to the interpretation of the analysis, writing, and approval of the manuscript. Both authors agreed and approved the submitted version.

## Funding

This project was supported by the Office of Research and Development of the University of Botswana through Internal Research Round 39 Funding Award—R1250.

## Conflict of Interest

The authors declare that the research was conducted in the absence of any commercial or financial relationships that could be construed as a potential conflict of interest.

## Publisher's Note

All claims expressed in this article are solely those of the authors and do not necessarily represent those of their affiliated organizations, or those of the publisher, the editors and the reviewers. Any product that may be evaluated in this article, or claim that may be made by its manufacturer, is not guaranteed or endorsed by the publisher.
